# Circadian-Coupled Genes Expression and Regulation in HIV-Associated Chronic Obstructive Pulmonary Disease (COPD) and Lung Comorbidities

**DOI:** 10.3390/ijms24119140

**Published:** 2023-05-23

**Authors:** Kingshuk Panda, Srinivasan Chinnapaiyan, Md. Sohanur Rahman, Maria J. Santiago, Stephen M. Black, Hoshang J. Unwalla

**Affiliations:** 1 Department of Immunology and Nanomedicine, Herbert Wertheim College of Medicine, Florida International University, 11200 SW 8th Street, Miami, FL 33199, USA; kpand014@fiu.edu (K.P.); schinnap@fiu.edu (S.C.); mdsrahma@fiu.edu (M.S.R.); msant206@fiu.edu (M.J.S.); 2 Department of Cellular Biology & Pharmacology, Herbert Wertheim College of Medicine, Florida International University, 11200 SW 8th Street, Miami, FL 33199, USA; stblack@fiu.edu

**Keywords:** HIV, COPD, circadian genes, circadian clocks, lung inflammation, pulmonary comorbidities

## Abstract

People living with HIV (PLWH) have an elevated risk of chronic obstructive pulmonary disease (COPD) and are at a higher risk of asthma and worse outcomes. Even though the combination of antiretroviral therapy (cART) has significantly improved the life expectancy of HIV-infected patients, it still shows a higher incidence of COPD in patients as young as 40 years old. Circadian rhythms are endogenous 24 h oscillations that regulate physiological processes, including immune responses. Additionally, they play a significant role in health and diseases by regulating viral replication and its corresponding immune responses. Circadian genes play an essential role in lung pathology, especially in PLWH. The dysregulation of core clock and clock output genes plays an important role in chronic inflammation and aberrant peripheral circadian rhythmicity, particularly in PLWH. In this review, we explained the mechanism underlying circadian clock dysregulation in HIV and its effects on the development and progression of COPD. Furthermore, we discussed potential therapeutic approaches to reset the peripheral molecular clocks and mitigate airway inflammation.

## 1. Introduction

Human Immunodeficiency Virus (HIV) is a significant global public health issue, even in the cART era. The World Health Organization (WHO) estimates that around 38.4 million people will live with HIV by the end of 2023, whereas 25.6 million people live in the African region [1]. The CDC HIV care continuum shows that only 53% of PLWH are virally suppressed in the United States [2].

Several HIV-associated lung comorbidities are prevalent in PLWH. While AIDS-associated comorbidities such as Pneumocystis infections have declined, non-AIDS comorbidities such as pulmonary arterial hypertension, bacterial pneumonia, and COPD are a significant concern for morbidity and mortality in people living with HIV in the cART era [3]. 

The mechanisms underlying non-AIDS pulmonary disease in HIV are poorly understood. HIV-infected patients develop COPD, even when they are compensated for smoking. Chronic lung diseases, such as chronic obstructive pulmonary disease and asthma, have a substantial public health burden, primarily COPD, the third leading cause of death globally. The onset of obstructive lung disease in PLWH is almost a decade earlier than in their non-HIV counterparts. Smoking exacerbates COPD, which is a concern as ~60% of people living with HIV are smokers [4]. Hence, respiratory therapies must be developed to treat the increased number of PLWH who manifest COPD [5]. Under this circumstance, it is imperative to understand the pathophysiology underlying the comorbidity of COPD in PLWH to develop a therapeutic approach targeting these underlying mechanisms. 

There are multiple hypotheses that propose several explanations for the pathogenesis of COPD in HIV-infected patients. Chronic lung inflammation is a hallmark of COPD, and there are multiple pathways that individually and additively promote lung inflammation in COPD. There is the possibility that these processes could coincide at the same time [4]. Even PLWH on cART show an increased incidence of unrelated AIDS pulmonary complications compared to their non-infected counterparts. Interestingly, HIV and COPD are associated with an increase in CD8+ lymphocytes, which have been shown to play a role in lung inflammation [4]. COPD exacerbations also demonstrate a circadian disruption with increased exacerbations at night (nocturnal desaturation) or early morning hours, possibly due to circadian dysregulation of mucus physiology and lung inflammation [6]. 

Circadian rhythms are 24 h cycles that are part of the body’s internal clock, which maintains physical, mental, and behavioral changes [7]. The circadian system is composed of a hierarchy of oscillators that functions in different contexts. The circadian clock can be divided into two components: the central clock and the peripheral clock. The central clock is in the suprachiasmatic nucleus (SCN) of the hypothalamus in the brain, which receives and is regulated by light. The peripheral clocks are biological clocks found in organs and tissues. The peripheral clocks control several physiological processes. The hypothalamic suprachiasmatic nucleus (SCN) is considered the “master” clock and serves to synchronize peripheral clocks. However, the peripheral clock can be dysregulated by environmental stimuli and metabolic status. The central and peripheral circadian clocks are both regulated by feedback loops. A reciprocal relationship exists between molecular clock disruption and immune/inflammatory responses [8]. 

PLWH demonstrated aberrant circadian rhythmicity [9,10], specifically, an alteration in the circadian rhythm of various genes involved in inflammation, oxidative stress, and immune function [11]. Studies have reported that this alteration can contribute to the development and progression of several conditions associated with HIV, such as chronic inflammation [12,13]. There is a link between circadian gene expression and severe lung inflammation, particularly in PLWH. Circadian genes constitute the core clock genes such as BMAL1, CLOCK, PER, and CRY, and clock output genes such as RevERBα, RORA, and CCG [9]. In mammals, the BMLA1: CLOCK activator complex binds to E-box elements on the promoters of circadian-regulated genes, including the expression of clock output genes, REV-ERBs α/β, and negative regulators PER and CRY. Consequently, these genes suppress the expression of BMAL1 and CLOCK, establishing a negative feedback loop through transcription/translation and maintaining the expression of circadian genes. CLOCK acetylates H3 and H4, making the chromatin epigenetically favorable for the transcription of downstream genes. CLOCK also acetylates BMAL1 and PER2 (recruited by Ac-BMAL1). Ac-PER2 recruits SIRT1, which deacetylates histones, PER2, and BMAL1. Consequently, PER2 reverts to the repressive chromatin state. PER2 dissociation leads to the dissociation of SIRT1, and the cycle is repeated [14]. Several recent studies support that the HIV-1 Trans-activator of transcription (Tat) protein affects circadian rhythmicity by interfering with the circadian clock in PLWH and is a possible factor in HIV-mediated COPD [9,15,16]. In this review, we will discuss the role of circadian gene dysregulation in HIV and COPD and identify a possible link between HIV proteins, lung circadian clock dysregulation, and lung inflammation. We will further discuss possible therapeutic strategies to reset the lung circadian clock to mitigate lung inflammation in HIV-associated COPD.

## 2. Spectrum of HIV-Mediated Lungs Complications

**Figure 1:**
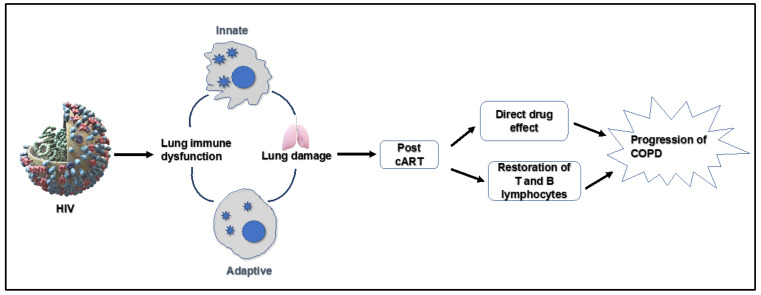
Potential mechanisms of antiretroviral-mediated lung damage in HIV.

PLWH demonstrate significant lung comorbidities. Chronic inflammation, aberrant immune activation and regulation, endothelial dysfunction, oxidative stress, and pulmonary systemic dysbiosis mediate COPD development. Refs. [17,18,19] report that lung inflammation is a hallmark of COPD, as shown in Figure 1. HIV infection can cause significant changes in pulmonary immune function, which can lead to increased susceptibility to respiratory infections and other lung diseases [20]. HIV can profoundly affect innate and adaptive immune responses in the lungs. Innate immunity involves the recognition of pathogen-associated molecular patterns, which activate many cells along with a wide range of pro-inflammatory pathways. HIV can affect various aspects of the innate immune system in the lung, ranging from the airway epithelium and surfactant proteins to alveolar macrophages, dendritic cells, and natural killer cells. HIV can infect bronchial epithelial cells as these cells express the HIV receptor CD4, C-C chemokine receptor type 5 (CCR5), and C-X-C chemokine receptor type 4 (CXCR-4) [21]. A study by Chinnapaiyan et al., 2017 reported that HIV infection suppresses tracheobronchial mucociliary clearance and may predispose HIV-infected patients to recurrent lung infections, pneumonia, and chronic bronchitis [22]. Studies have demonstrated that HIV can significantly affect both the bronchial and alveolar epithelium, and could represent a mechanism by which HIV infection renders individuals susceptible to lung injury [23]. 

HIV also targets the adaptive immune system in the lungs through chronic immune activation and immune suppression, as well as reduced proliferative and cytotoxic capacity. These effects lead to the progressive loss of T cell functionality, including a decreased cytokine response to antigenic stimulation and B cell activation, resulting in the impairment of long-term serologic immunity [24]. 

### 2.1. Chronic Obstructive Pulmonary Disease (COPD)

Cigarette smoking exacerbates the development and progression of COPD [25]. This is concerning because a significant number of PLWH are smokers [26,27]. Additionally, HIV infection has been associated with accelerated emphysema and airway obstruction. 

Several studies examined the respiratory symptoms and pulmonary function in HIV patients treated with cART [28,29,30]. The frequency of respiratory symptoms and airway obstruction was associated with age, smoking history, and pneumonia in 31% of participants. The mechanism by which HIV leads to chronic lung inflammation is still not completely understood. Data from various retrospective studies have shown that PLWH have a higher risk of COPD and potentially worse asthma outcomes than the non-infected population [31]. More recently, study groups have used different non-invasive lung assessment tests to determine lung function, such as the single-breath diffusing capacity of carbon monoxide (Dl_CO_), fractional exhaled nitric oxide (FeNO), and lung structure determination using chest computed tomography (CT) [32,33]. 

The DLCO assessments confirmed that PLWH have diminished gas exchange efficiency compared to HIV-negative controls [33]. FeNO-based studies also reported that PLWH have increased Th2 inflammation in the lungs [33]. Interestingly, CT chest studies also showed that PLWH are at a higher risk of structural lung tissue destruction, even among non-smokers [33]. Recently, Besutti and colleagues from Modena/Italy performed a single-center study comparing 145 non-smoker PLWH to 75 matched controls [34]. The study reported that PLWH had a four-fold higher risk of emphysema than non-HIV-infected subjects. An ongoing clinical study analyzing the microbiome and TH17-mediated inflammation in PLWH in Uganda (NCT05223114) has demonstrated that PLWH who also had COPD were significantly enriched in specific microbes, with the microbiome dependent more on HIV than on COPD. These studies suggest that HIV may promote a unique airway microbiome that drives inflammation and COPD development/progression genera among PLWH.

### 2.2. Asthma

Asthma is another obstructive lung disease that is prevalent in PLWH. Bronchial hyperresponsiveness (BHR) is a classic feature of asthma. People with HIV have an increased incidence of respiratory symptoms, elevated IgE levels, more response to bronchoprovocation, and a higher incidence of asthma than seronegative controls. A study of 1202 HIV-positive youth found that asthma incidence increased significantly from 2008 to 2014 compared to 2004–2007 [35]. In a study of HIV-positive adults, ~10–20% had been told by their doctor that they had asthma. Another report suggested that in Uganda, asthma was higher in PLWH. In people without HIV, HIV interacts synergistically with other known asthma risk factors [36]. A study by Barton JA et al., 2016 performed a cross-sectional study of 121 HIV-infected individuals with asthma phenotype for pulmonary function testing and chest CT scans to measure airway wall thickness, adipose tissue volumes, and biomarkers [37]. The study reported that airway wall thickness, as measured on a CT scan, is associated with subcutaneous adipose volume, lower peripheral adiponectin, an anti-inflammatory adipokine, and a higher level of CRP in PLWH.

The exact mechanism by which HIV influences asthma’s development or its clinical course remains uncertain. Several reports have suggested that ART use is associated with an increased risk of asthma [38,39]. In contrast, chemokines released by CD8+ T cells and HIV suppressive factors are associated with a greater risk [40]. These chemokines have been associated with asthma-related airway inflammation in the HIV-negative population, suggesting that chronic HIV infection may stimulate an immune response that could contribute to asthma pathogenesis [41]. HIV-seropositive individuals with BHR had significantly increased positive skin testing and elevated serum IgE levels compared to those without BHR [42]. This suggests a potential correlation between respiratory symptoms, IgE levels, and BHR in people with HIV infection, which is consistent with asthma.

### 2.3. Pulmonary Hypertension

Infection with HIV increases the risk of pulmonary hypertension. It is a severe and potentially life-threatening condition characterized by elevated blood pressure in the arteries that supply blood to the lungs. There are different types of PH, but one of the most common forms of PH associated with HIV is pulmonary arterial hypertension (PAH). PAH is characterized by the narrowing and stiffening of the pulmonary arteries, which increases pressure in the lungs. The exact mechanism underlying HIV-mediated PAH is still unknown. HIV proteins, including envelope glycoprotein-120, HIV Env, and Transactivator of transcription (Tat), can damage endothelial cells and cause inflammation, resulting in pulmonary vascular remodeling [43,44]. Studies have reported that HIV-1 Nef, a broad-spectrum adaptor protein, may affect HIV-infected and un-infected pulmonary vascular cells [45]. In addition, HIV infection causes chronic inflammation and immune activation, which can damage blood vessels in the lungs and contribute to the development of pulmonary hypertension.

### 2.4. Lung Cancer

People living with HIV are more likely to develop lung cancer than the general population [46,47]. The increased risk is related to several factors, including higher smoking rates, chronic inflammation, and immune dysfunction [47]. Smoking is a significant risk factor for lung cancer, and people living with HIV have been shown to have higher rates of smoking than the general population. Another study found that HIV-positive individuals with a low CD4 count, indicating more advanced immune suppression, had a higher risk of lung cancer than those with a higher CD4 count. Hleyhel et al. found that HIV-infected persons who did not recover their CD4 count to at least 500 cells/mm^3^ were at risk of lung cancer [48]. 

## 3. Molecular Clock Gene Involvement in Pulmonary Complications

The circadian clock in the lungs is critical for optimizing the organization of cellular functions and responses to environmental stimuli. Any significant change in the pattern of circadian genes, known as circadian disruption, can affect downstream gene expression and could be implicated in chronic diseases. Rhythms of clock genes have been reported in the lungs, which include bronchial epithelial cells. Studies have reported that healthy individuals usually have a strong daily pattern in their lung function, showing the highest levels around noon (12.00 h) and the lowest levels in the early morning (04.00 h). This increase in lung function during the early morning can overlap with exacerbations of COPD/asthma in susceptible individuals [49]. Numerous studies have shown the role of clock dysfunction in pulmonary physiology and pathology, particularly in response to pro-inflammatory cytokine mediators such as CS. COPD is generally characterized by alveolar epithelial injury and persistent airway inflammation. Patients with obstructive airway disease, including COPD, develop severe exacerbations, specifically at night and in the early morning hours when lung function is at its lowest [50]. The effect of the virus-induced COPD exacerbations on clock function in the lungs remains unclear. Nevertheless, there is a link between the circadian clock and the decline in lung function [51,52]. Circadian rhythms are intrinsic biological oscillations with a period near 24 h driven in mammals by the circadian timing system. Light stimulation serves as zeitgebers (timing cues) to the brain’s suprachiasmatic nucleus (SCN), which is the central pacemaker synchronizing peripheral clocks. Circadian rhythms are generated due to the actions of an inter-regulated system of circadian transcription factors called clock genes [53]. The circadian clock is cell autonomous, and a “transcription-translation” feedback loop controls its operation. Bmal1 and Clock are the two principal transcription factors that drive this complex molecular clock in mammals. The Bmal1: clock activator complex regulates the expression of the period (Per1-3) and cryptochrome (Cry1-2) genes [54,55,56]. After translation, Per and Cry form heterodimers that are phosphorylated and translocated back to the nucleus, where they repress their transcription by blocking the activity of the Bmal1: Clock complex. Bmal1: Clock and the Per and Cry complex are broadly involved in negative feedback regulation. Interestingly, the nuclear receptors REV-ERBα/β, RORα, and CCG regulate the transcription of clock genes and other target genes maintaining the circadian expression [57]. 

Studies have reported that, in the lungs of mice with chronic CS exposure, the expression of Bmal1, REV-ERBα (Nr1d1), and Per1 decreased, and the express process of Per1 and Per2 may also change. Moreover, clinical studies have found a decrease in the number of Bmal1 in COPD patients’ lungs. It is believed that such dysfunction partially results from the clock’s transcription regulated by acetylated and degraded Bmal1 and Per2. Several studies have indicated that Sirtuin 1 (SIRT1), a metabolic NAD+-dependent protein/histone deacetylase that regulates proinflammatory mediators by deacetylating histone and non-histone proteins, is a master regulator associated with the lungs of smokers with COPD. Under normal conditions, SIRT1 affects clock function by binding to the CLOCK: BMAL1 complex and deacetylating BMAL and PER2 proteins [58,59]. Recently, Li. et al., 2022 reported a comparative in vitro study to understand the role of circadian protein BMAL1 and CLOCK in plasma samples of non-smokers, smokers, and patients with COPD [60]. The study reported several observations. First, lower Bmal1 and Clock expression was observed in the plasma of patients with COPD; second, cigarette smoke exposure (CSE) caused the inhibition of Bmal1/Clock expression; and third, CSE increased the expression of cell senescence in human bronchial epithelial cells by interfering with MAPK pathways. Another study by Sundar et al., 2017 showed a significant reduction in REV-ERBα in small airway epithelial cells taken from patients with COPD [61]. Studies from animal models highlight the extent to which these molecular clocks are essential, which regulate the fundamental aspects of immune-inflammatory responses [62,63]. Recent evidence suggests that the molecular clock is responsible for regulating fundamental aspects of the immune-inflammatory responses such as Toll-like receptor 9 (TLR9) signaling and repressing chemokine (C-C motif) ligand 2 (CCL2) expression [64,65]. The clock protein and nuclear heme receptor REV-ERBα attenuate the activation of the IL-6 receptor [66]. In addition, it has been reported that REV-ERBα binds with nuclear factor Kappa beta (NF-κB) and promotes oxidative stress and inflammation [67,68]. 

Similarly, a study by Chen HC et al., 2021 showed altered expression of circadian clock genes in patients with bronchial asthma and downregulated PER3 in patients with nocturnal symptoms [69]. In asthmatic patients, the expressions of BMAL1, CKlε, CLOCK, CRY1, CRY2, and PER1 were significantly lower in the patients with nocturnal symptoms than in those without nocturnal symptoms. The binary logistic regression confirmed the association of BMAL1, CKlε, PER3, and TIM as independent risk factors for bronchial asthma. 

## 4. Pathogenesis of HIV and Circadian Disruption

According to the CDC-HIV care continuum, a very low percentage of PLWH are virally suppressed in the United States; as a result, many PLWH will have replicating viruses [2]. The cART can only suppress HIV but does not eradicate it due to the existence of latently infected anatomical reservoirs [70,71]. Replication persists in these reservoirs despite suppressive cART [72,73]. The lungs are anatomical reservoirs of HIV [74,75,76], with alveolar macrophages, intrapulmonary lymphocytes [75,76], and even bronchial epithelial cells serving as reservoirs [22,77,78,79,80]. Several studies have reported that HIV can cause lung damage and inflammation that may contribute to the development of COPD. The risk of COPD is exceptionally high in people with HIV who smoke or have a history of AIDS-related infections. The pathogenesis of lung-related co-morbidities in people living with HIV is still not fully understood. Still, it is believed to involve a complex interplay of factors, including chronic inflammation, immune dysfunction, and environmental exposure. Some evidence suggests that circadian disruption may play a role in the pathogenesis of COPD and other lung-related co-morbidities in people living with HIV. 

A recent study has demonstrated that the “silent majority” of the HIV reservoir is transcriptionally active [81,82], providing a source of HIV proteins such as Tat in the airway. Malone et al., 1992 first reported that PLWH show a circadian pattern of progressive loss of CD4^+^ T lymphocytes between morning and evening [83]. Previous studies have demonstrated a correlation between the concentration of Tat protein in HIV patients’ blood, patients’ sleep quality, and melatonin concentration. Wang T. et al., 2014 found that the sleep quality of patients with HIV/AIDS was affected by an altered circadian rhythm which correlates with the cerebrospinal HIV Tat Protein concentration [9]. Likewise, Lee et al., 2015 investigated the relationship between circadian gene polymorphism and sleep patterns in adults with HIV [84]. These results show that specific polymorphisms in circadian regulation genes, such as CLOCK, CRY1, PER1, PER2, and PER3 are associated with sleep disruption and duration, circadian phase, and rhythm in PLWH. This study extends the evidence that genetic factors may regulate sleep patterns in individuals with HIV. 

HIV infection depends on the activated state of CD4+ T cells, as the virus cannot replicate in resting T cells. T-cell activation enhances viral transcription by activating various transcription factors, especially the nuclear factor kB (NF-κB). NF-κB is a critical regulator of HIV transcription and a master mediator of HIV escape from latency. HIV inhibits the activation of NF-κB, which is involved in regulating various immune responses. Under normal conditions, SIRT1, an NAD+-dependent protein acetylase, controls the transcriptional activity of the NF-κB complex by deacetylating lysine 310 in p65 of NF-κB. Upon HIV infection, HIV Tat directly interacts with the deacetylase domain of SIRT1 and blocks the ability of SIRT1 to deacetylate lysine 310 in the p65 subunit of NF-κB [85]. As a result, NF-κB loses its anti-apoptotic function and immune cell hyperactivation is observed in HIV-infected individuals. Clark JP et al., 2005 first reported the impact of HIV Tat on circadian rhythms in the body [15]. The results showed that Tat alters circadian rhythms through the light entrainment pathway, suggesting that Tat may disrupt the normal regulation of circadian rhythms in PLWH.

**Figure 2:**
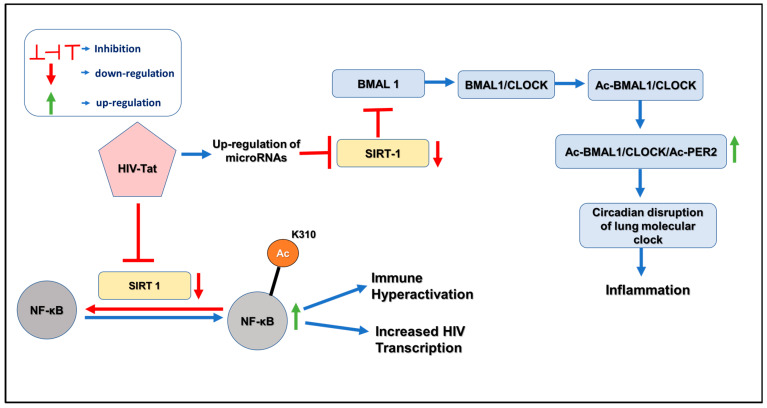
Interplay between SIRT1 and Circadian gene disruption by HIV Tat in the lungs.

Kwon HS et al., 2008 showed that HIV Tat directly interacts with the SIRT1 and blocks the deacetylase domain of SIRT1. Given that SIRT1 deacetylation of NF-kβ is involved in suppressing NF-κβ regulation, this results in the hyperactivation of NF-κβ responsive genes and contributes to immune cell hyperactivation in HIV-infected individuals (Figure 2). SIRT1 is the master regulator of the circadian clock due to its ability to drive the deacetylation of Per2 and BMAL1 [14]. In HIV-infected patients, HIV Tat suppresses SIRT1 through an unknown mechanism, increasing Ac-BMAL1 and Ac-PER2. As a result, it disrupts the lung molecular clock and, consequently, increases inflammation. Recently, a study by Bordoni V et al., 2020 investigated the expression of the Per2 gene in hematopoietic progenitor cells in individuals with chronic infection [86]. The results showed that Per2 gene expression was upregulated in these cells during chronic HIV infection due to the downregulation of deacetylase SIRT1. 

## 5. Aberrant microRNAome Mediated Dysregulation of Clock Genes in HIV-Mediated COPD

miRNAs are small non-coding RNA (20–22 nucleotides) molecules that regulate gene expression by binding to mRNA (messenger RNA) and preventing protein translation or degradation [87]. Most miRNAs are transcribed from DNA sequences into primary miRNAs (pri-miRNAs) and processed into precursor (pre-miRNAs) and mature miRNAs. miRNAs also fundamentally mediate biological mechanisms that regulate mRNA expression post-transcriptionally, affecting cellular events, including metabolism, growth, cell differentiation, development, apoptosis, inflammation, and cell signaling [88]. Since the complementary base sequences between miRNAs and their target mRNA are somewhat redundant, a single miRNA can simultaneously regulate tens to hundreds of genes. The human genome has been reported to encode nearly 1917 annotated hairpin precursors and 2654 mature sequences [89]. miRNAs have been shown to regulate the expression of up to 60% of mammalian and 90% of human protein-coding genes [90,91]. As miRNAs are only partially complementary to their target mRNAs, each miRNA is estimated to be capable of regulating the expression of up to 200 genes [92,93]. 

Numerous diseases, including lung disease and solid and hematological cancers, have been linked to the pathophysiology of miRNA dysregulation. Interestingly, recent studies have suggested that miRNAs may be involved in the pathogenesis of HIV-mediated COPD. miRNAs play a key role in modulating gene expression, and it is evidenced that numerous miRNAs are involved in the pathology of COPD [94]. Comparable to non-infectious conditions, miRNAs affect host and virus interaction in various ways. HIV-1 mainly infects human CD4+ T cells by recognizing the CD4+ receptor on the cell surface. HIV-1 replication within the host cell is regulated by various host factors, including miRNAs, which may target viral mRNA directly to regulate the expression of host proteins that HIV-1 hijacks for its replication [95]. In addition, miRNAs are linked with a possible susceptibility to HIV infection in monocytes and macrophages. Furthermore, the viral genome may produce virally encoded miRNAs that modulate viral RNAs and cellular mRNAs. 

Circadian genes provide instructions for producing proteins that affect the regulation of the body’s daily rhythms, also known as the circadian clock. Examples of circadian genes are Clock, Bmal, Per1, Per2, Cry1, and Cry2 [96]. It is well-documented that circadian rhythms can lead to oxidative stress, inflammation, and other cellular processes contributing to COPD [97]. These circadian genes regulate miRNAs or are regulated by miRNAs using different molecular mechanisms and factors. miRNAs play a crucial role in repressing mRNA translation or penetrating the mRNA of the gene of interest and have been demonstrated to be involved in the regulation of clock gene expression in angiogenesis [98]. 

An earlier study by Cheng HY et al. first reported that two brain-specific miRNAs, miR-219 and miR-132, play a critical role in modulating the circadian complex CLOCK/BMAL1 in the suprachiasmatic nucleus [99]. According to Shende et al., miR-142-3p and miR-494 precisely target the Bmal1 gene and regulate circadian expression in the suprachiasmatic nuclei (SCN) [100,101]. Studies have demonstrated that miR-142-3p is crucial for the negative feedback regulation of the molecular clock, along with the suppression of BMAL1 and SIRT1 genes by miR-142-3p and miR-142-5p, respectively [102,103]. CLOCK regulates miR-142 expression, which exhibits circadian rhythmicity by binding upstream of the miR-142 E-box region [30,31]. As a result, miR-142 acts as a core clock-controlled miRNA. Studies have already reported that miR-142 is upregulated in HIV and SIVE encephalitis [103]. Similarly, HIV Tat up-regulated the expression of miR-34a-5p and decreased the SIRT1 protein expression [104]. It has been shown that several microRNAs specifically target the PER genes of the molecular clock. It is well established that miR-34a-5p also targets CRY1, PER1, and PER2 in the molecular clock [105,106,107]. Similar evidence has been found that miR-24 and miR-29b decrease PER2 and PER3, respectively [108,109]. Although, till now, there is no report regarding the upregulation of miR-24 and miR-29b during HIV infection. Evidence shows that PER gene inhibition shortens the circadian period and alters the circadian cycle.

The miR-17-92 cluster is widely expressed in endothelial cells, and miR-92a, in particular, is thought to play a role in angiogenesis by targeting the mRNAs of proangiogenic proteins such as integrin a5 [110]. It has been found that miR-92a expression increases 5- to 10-fold in CD34+ cells, and this miR-92a potentially regulates the clock gene Per2 [111]. It has also been demonstrated that miR-17-5p directly controls the CLOCK gene [112]. Interestingly, reciprocal regulation is found in that the CLOCK protein binds to the miR-17 promoter region directly and activates the expression of miR-17-5p, which then binds to the 3’ UTR of CLOCK and downregulates its expression [112]. However, a study conducted by Triboulet R et al., 2007 reported the downregulation of the miR-17-92 cluster upon HIV infection [113]. 

A microRNA cluster called the miRNA-192/194 cluster, discovered by the research group of Nagel et al., 2009, directly controls the core elements of the circadian clock [114]. Using a target-based screening approach, they showed that the endogenously expressed miR-192/194 cluster acts as a prospective regulator and inhibitor of the whole Period gene family (PER1, PER2, and PER3). The target sites for miR-192/miR-194 are radially available on the 3′-UTR of all PER genes. In a study conducted by Na et al., 2009 it was observed that miR-181d and miR-191 exhibited an inversely correlated circadian rhythm in the mouse liver [115]. Another study by Witwer KW et al., 2012 reported the upregulation of miR-181b/d upon HIV infection [116].

## 6. Discussion

Previous reports have shown that cigarette smoke and COPD are associated with decreased SIRT1 and other clock genes as a consequence of chronic lung inflammation. 

HIV reservoirs in the lungs express HIV proteins such as Tat despite the suppressive cART-promoting expression of pro-inflammatory cytokines due to NK-κβ activation. NF-κβ activation is a consequence of Tat-mediated SIRT1 suppression, leading to a loss of deacetylation of NF-κβ subunits p65 and p53, consequently leading to increased secretion of proinflammatory cytokines. 

However, the underlying pathophysiological mechanism by which HIV Tat disrupts the circadian clock, ultimately promoting chronic lung inflammation, remains unknown. Recent evidence suggests that non-coding RNA, specifically microRNAs, are essential for regulating circadian genes [107,117,118,119,120,121]. A few studies also revealed that miR-142-5p and miR-34a-5p play an essential role in the regulation of the molecular clock by modulating the expression of SIRT1 [103,122], whereas miR-142-3p can control the regulation of BMAL1 [100,102]. Given that HIV Tat dysregulates multiple microRNAs, including miR-142-5p [123], future research should focus on the roles of post-transcriptional gene silencing by microRNAs as intermediates in clock dysregulation in smokers and PLWH.

## 7. Conclusions

The Circadian clock regulates gene expression in response to the body’s internal circadian rhythm, which governs the daily rhythmic changes in physiological processes. In PLWH, it is thought that dysregulation of the circadian clock may play an important role in COPD pathogenesis. Lung diseases such as COPD have become more prevalent in PLWH despite suppressive cART. This review provides a first peak at the role of circadian gene dysregulation in HIV and COPD. It identifies a possible link between HIV proteins, lung circadian clock dysregulation, and lung inflammation. Understanding this mechanism will help us to develop therapeutic strategies to reset the lung circadian clock to mitigate lung inflammation in HIV-associated COPD.

## Data Availability

Not applicable.

## References

[1] Taylor S.W., McKetchnie S.M., Batchelder A.W., Justice A., Safren S.A., O’Cleirigh C. (2022). Chronic pain and substance use disorders among older sexual minority men living with HIV: Implications for HIV disease management across the HIV care continuum. AIDS Care.

[2] CDC (2019). Undertanding the HIV Care Continuum. https://www.cdc.gov/hiv/pdf/library/factsheets/cdc-hiv-care-continuum.pdf.

[3] Bigna J.J., Kenne A.M., Asangbeh S.L., Sibetcheu A.T. (2018). Prevalence of chronic obstructive pulmonary disease in the global population with HIV: A systematic review and meta-analysis. Lancet Glob. Health.

[4] Morris A., George M.P., Crothers K., Huang L., Lucht L., Kessinger C., Kleerup E.C. (2011). HIV and chronic obstructive pulmonary disease: Is it worse and why?. Proc. Am. Thorac. Soc..

[5] Lambert A.A., Kirk G.D., Astemborski J., Mehta S.H., Wise R.A., Drummond M.B. (2015). HIV infection is associated with increased risk for acute exacerbation of COPD. JAIDS J. Acquir. Immune Defic. Syndr..

[6] Comas M., Gordon C.J., Oliver B.G., Stow N.W., King G., Sharma P., Ammit A.J., Grunstein R.R., Phillips C.L. (2017). A circadian based inflammatory response–implications for respiratory disease and treatment. Sleep Sci. Pract..

[7] Hunter F.K., Butler T.D., Gibbs J.E. (2022). Circadian rhythms in immunity and host-parasite interactions. Parasite Immunol..

[8] Sundar I.K., Yao H., Sellix M.T., Rahman I. (2015). Circadian molecular clock in lung pathophysiology. Am. J. Physiol.-Lung Cell. Mol. Physiol..

[9] Wang T., Jiang Z., Hou W., Li Z., Cheng S., Green L., Wang Y., Wen X., Cai L., Clauss M. (2014). HIV T at protein affects circadian rhythmicity by interfering with the circadian system. HIV Med..

[10] Swoyer J., Rhame F., Hrushesky W., Sackett-Lundeen L., Sothern R., Gale H., Haus E. (1990). Circadian rhythm alteration in HIV infected subjects. Prog. Clin. Biol. Res..

[11] Duncan M.J., Bruce-Keller A.J., Conner C., Knapp P.E., Xu R., Nath A., Hauser K.F. (2008). Effects of chronic expression of the HIV-induced protein, transactivator of transcription, on circadian activity rhythms in mice, with or without morphine. Am. J. Physiol.-Regul. Integr. Comp. Physiol..

[12] Butler T.D., Mohammed Ali A., Gibbs J.E., McLaughlin J.T. (2023). Chronotype in patients with immune-mediated inflammatory disease: A systematic review. J. Biol. Rhythm..

[13] Rijo-Ferreira F., Takahashi J.S. (2022). Circadian Rhythms in Infectious Diseases and Symbiosis. Seminars in Cell & Developmental Biology.

[14] Belden W.J., Dunlap J.C. (2008). SIRT1 is a circadian deacetylase for core clock components. Cell.

[15] Clark J., Sampair C.S., Kofuji P., Nath A., Ding J.M. (2005). HIV protein, transactivator of transcription, alters circadian rhythms through the light entrainment pathway. Am. J. Physiol.-Regul. Integr. Comp. Physiol..

[16] Chang C.C., Naranbhai V., Stern J., Roche M., Dantanarayana A., Ruian K., Tennakoon S., Solomon A., Rebecca H., Hartogensis W. (2018). Variation in cell associated unspliced HIV RNA on antiretroviral therapy is associated with the circadian regulator BMAL-1. AIDS.

[17] van Eeden S.F., Hogg J.C. (2020). Immune-modulation in chronic obstructive pulmonary disease: Current concepts and future strategies. Respiration.

[18] Bhat T.A., Panzica L., Kalathil S.G., Thanavala Y. (2015). Immune dysfunction in patients with chronic obstructive pulmonary disease. Ann. Am. Thorac. Soc..

[19] Kayongo A., Robertson N.M., Siddharthan T., Ntayi M.L., Ndawula J.C., Sande O.J., Bagaya B.S., Kirenga B., Mayanja-Kizza H., Joloba M.L. (2023). Airway microbiome-immune crosstalk in chronic obstructive pulmonary disease. Front. Immunol..

[20] Cribbs S.K., Crothers K., Morris A. (2020). Pathogenesis of HIV-related lung disease: Immunity, infection, and inflammation. Physiol. Rev..

[21] Alexandrova Y., Costiniuk C.T., Jenabian M.-A. (2022). Pulmonary immune dysregulation and viral persistence during HIV infection. Front. Immunol..

[22] Chinnapaiyan S., Parira T., Dutta R., Agudelo M., Morris A., Nair M., Unwalla H. (2017). HIV infects bronchial epithelium and suppresses components of the mucociliary clearance apparatus. PLoS ONE.

[23] Fan X., Murray S.C., Staitieh B.S., Spearman P., Guidot D.M. (2021). HIV impairs alveolar macrophage function via MicroRNA-144-induced suppression of Nrf2. Am. J. Med. Sci..

[24] Charles T.P., Shellito J.E. (2016). Human immunodeficiency virus infection and host defense in the lungs. Seminars in Respiratory and Critical Care Medicine.

[25] Antoniou T., Yao Z., Raboud J., Gershon A.S. (2020). Incidence of chronic obstructive pulmonary disease in people with HIV in Ontario, 1996–2015: A retrospective population-based cohort study. Can. Med. Assoc. Open Access J..

[26] Rahmanian S., Wewers M.E., Koletar S., Reynolds N., Ferketich A., Diaz P. (2011). Cigarette smoking in the HIV-infected population. Proc. Am. Thorac. Soc..

[27] Unwalla H.M.A. (2015). Trachebronchial Mucociliary Dysfunction in HIV. J. Neuroimmune Pharm..

[28] Lifson A.R., Neuhaus J., Arribas J.R., van den Berg-Wolf M., Labriola A.M., Read T.R., Group I.S.S. (2010). Smoking-related health risks among persons with HIV in the Strategies for Management of Antiretroviral Therapy clinical trial. Am. J. Public Health.

[29] Gingo M.R., George M.P., Kessinger C.J., Lucht L., Rissler B., Weinman R., Slivka W.A., McMahon D.K., Wenzel S.E., Sciurba F.C. (2010). Pulmonary function abnormalities in HIV-infected patients during the current antiretroviral therapy era. Am. J. Respir. Crit. Care Med..

[30] van Riel S.E., Klipstein-Grobusch K., Barth R.E., Grobbee D.E., Feldman C., Shaddock E., Stacey S.L., Venter W.D., Vos A.G. (2021). Predictors of impaired pulmonary function in people living with HIV in an urban African setting. South. Afr. J. HIV Med..

[31] Zifodya J.S., Triplette M., Shahrir S., Attia E.F., Akgun K.M., Hoo G.W.S., Rodriguez-Barradas M.C., Wongtrakool C., Huang L., Crothers K. (2021). A cross-sectional analysis of diagnosis and management of chronic obstructive pulmonary disease in people living with HIV: Opportunities for improvement. Medicine.

[32] Kunisaki K.M. (2021). Recent advances in HIV-associated chronic lung disease clinical research. Curr. Opin. HIV AIDS.

[33] Modi P., Cascella M. (2020). Diffusing Capacity of the Lungs for Carbon Monoxide.

[34] Besutti G., Santoro A., Scaglioni R., Neri S., Zona S., Malagoli A., Orlando G., Beghè B., Ligabue G., Torricelli P. (2019). Significant chronic airway abnormalities in never-smoking HIV-infected patients. HIV Med..

[35] Mirani G., Williams P.L., Chernoff M., Abzug M.J., Levin M.J., Seage III G.R., Oleske J.M., Purswani M.U., Hazra R., Traite S. (2015). Changing trends in complications and mortality rates among US youth and young adults with HIV infection in the era of combination antiretroviral therapy. Clin. Infect. Dis..

[36] Kirenga B.J., Mugenyi L., de Jong C., Lucian Davis J., Katagira W., van der Molen T., Kamya M.R., Boezen M. (2018). The impact of HIV on the prevalence of asthma in Uganda: A general population survey. Respir. Res..

[37] Barton J.H., Ireland A., Fitzpatrick M., Kessinger C., Camp D., Weinman R., McMahon D., Leader J.K., Holguin F., Wenzel S.E. (2016). Adiposity influences airway wall thickness and the asthma phenotype of HIV-associated obstructive lung disease: A cross-sectional study. BMC Pulm. Med..

[38] Adrish M., Gomez G.R., Rodriguez E.C., Mantri N. (2019). Influence of HIV status on the management of acute asthma exacerbations. BMJ Open Respir. Res..

[39] Kendall C.E., Wong J., Taljaard M., Glazier R.H., Hogg W., Younger J., Manuel D.G. (2014). A cross-sectional, population-based study measuring comorbidity among people living with HIV in Ontario. BMC Public Health.

[40] Gingo M.R., Wenzel S.E., Steele C., Kessinger C.J., Lucht L., Lawther T., Busch M., Hillenbrand M.E., Weinman R., Slivka W.A. (2012). Asthma diagnosis and airway bronchodilator response in HIV-infected patients. J. Allergy Clin. Immunol..

[41] Fernandez-Botran R., Vega A.R., García Y., Tirumala C.C., Srisailam P., Raghuram A., Peyrani P., Furmanek S., Tella M.A., Ritzhentaler J.D. (2020). The elevated systemic cytokine levels in HIV patients are not associated with an elevated pulmonary cytokine environment. Cytokine.

[42] Poirier C.D., Inhaber N., Lalonde R.G., Ernst P. (2001). Prevalence of bronchial hyperresponsiveness among HIV-infected men. Am. J. Respir. Crit. Care Med..

[43] Kanmogne G.D., Primeaux C., Grammas P. (2005). HIV-1 gp120 proteins alter tight junction protein expression and brain endothelial cell permeability: Implications for the pathogenesis of HIV-associated dementia. J. Neuropathol. Exp. Neurol..

[44] Rusnati M., Presta M. (2002). HIV-1 Tat protein and endothelium: From protein/cell interaction to AIDS-associated pathologies. Angiogenesis.

[45] Sehgal P.B., Mukhopadhyay S., Patel K., Xu F., Almodóvar S., Tuder R.M., Flores S.C. (2009). Golgi dysfunction is a common feature in idiopathic human pulmonary hypertension and vascular lesions in SHIV-nef-infected macaques. Am. J. Physiol.-Lung Cell. Mol. Physiol..

[46] Shiels M.S., Pfeiffer R.M., Gail M.H., Hall H.I., Li J., Chaturvedi A.K., Bhatia K., Uldrick T.S., Yarchoan R., Goedert J.J. (2011). Cancer burden in the HIV-infected population in the United States. J. Natl. Cancer Inst..

[47] Silverberg M.J., Lau B., Achenbach C.J., Jing Y., Althoff K.N., D’Souza G., Engels E.A., Hessol N.A., Brooks J.T., Burchell A.N. (2015). Cumulative incidence of cancer among persons with HIV in North America: A cohort study. Ann. Intern. Med..

[48] Hleyhel M. (2014). Writing Committee of the Cancer Risk Group of the French Hospital Database on HIV. Risk of non-AIDS-defining cancers among HIV-1-infected individuals in France between 1997 and 2009: Results from a French cohort. Aids.

[49] Barnes P.J. (1985). Circadian variation in airway function. Am. J. Med..

[50] Angelis N., Porpodis K., Zarogoulidis P., Spyratos D., Kioumis I., Papaiwannou A., Pitsiou G., Tsakiridis K., Mpakas A., Arikas S. (2014). Airway inflammation in chronic obstructive pulmonary disease. J. Thorac. Dis..

[51] Sundar I.K., Ahmad T., Yao H., Hwang J.-W., Gerloff J., Lawrence B.P., Sellix M.T., Rahman I. (2015). Influenza A virus-dependent remodeling of pulmonary clock function in a mouse model of COPD. Sci. Rep..

[52] Ehlers A., Xie W., Agapov E., Brown S., Steinberg D., Tidwell R., Sajol G., Schutz R., Weaver R., Yu H. (2018). BMAL1 links the circadian clock to viral airway pathology and asthma phenotypes. Mucosal Immunol..

[53] Hastings M.H., Maywood E.S., Brancaccio M. (2019). The mammalian circadian timing system and the suprachiasmatic nucleus as its pacemaker. Biology.

[54] Takahashi J.S. (2017). Transcriptional architecture of the mammalian circadian clock. Nat. Rev. Genet..

[55] Matsumura R., Tsuchiya Y., Tokuda I., Matsuo T., Sato M., Node K., Nishida E., Akashi M. (2014). The mammalian circadian clock protein period counteracts cryptochrome in phosphorylation dynamics of circadian locomotor output cycles kaput (CLOCK). J. Biol. Chem..

[56] Parico G.C.G., Perez I., Fribourgh J.L., Hernandez B.N., Lee H.-W., Partch C.L. (2020). The human CRY1 tail controls circadian timing by regulating its association with CLOCK: BMAL1. Proc. Natl. Acad. Sci. USA.

[57] Mavroudis P.D., DuBois D.C., Almon R.R., Jusko W.J. (2018). Modeling circadian variability of core-clock and clock-controlled genes in four tissues of the rat. PLoS ONE.

[58] Rajendrasozhan S., Yang S.-R., Kinnula V.L., Rahman I. (2008). SIRT1, an antiinflammatory and antiaging protein, is decreased in lungs of patients with chronic obstructive pulmonary disease. Am. J. Respir. Crit. Care Med..

[59] Hwang J.-W., Sundar I.K., Yao H., Sellix M.T., Rahman I. (2014). Circadian clock function is disrupted by environmental tobacco/cigarette smoke, leading to lung inflammation and injury via a SIRT1-BMAL1 pathway. FASEB J..

[60] Li L., Zhang M., Zhao C., Cheng Y., Liu C., Shi M. (2022). Circadian clock gene Clock-Bmal1 regulates cellular senescence in Chronic obstructive pulmonary disease. BMC Pulm. Med..

[61] Lechasseur A., Jubinville É., Routhier J., Bérubé J.C., Hamel-Auger M., Talbot M., Lamothe J., Aubin S., Paré M.È., Beaulieu M.J. (2017). Exposure to electronic cigarette vapors affects pulmonary and systemic expression of circadian molecular clock genes. Physiol. Rep..

[62] Vitaterna M.H., King D.P., Chang A.-M., Kornhauser J.M., Lowrey P.L., McDonald J.D., Dove W.F., Pinto L.H., Turek F.W., Takahashi J.S. (1994). Mutagenesis and mapping of a mouse gene, Clock, essential for circadian behavior. Science.

[63] Deng W., Zhu S., Zeng L., Liu J., Kang R., Yang M., Cao L., Wang H., Billiar T.R., Jiang J. (2018). The circadian clock controls immune checkpoint pathway in sepsis. Cell Rep..

[64] Silver A.C., Arjona A., Walker W.E., Fikrig E. (2012). The circadian clock controls toll-like receptor 9-mediated innate and adaptive immunity. Immunity.

[65] Nguyen K.D., Fentress S.J., Qiu Y., Yun K., Cox J.S., Chawla A. (2013). Circadian gene Bmal1 regulates diurnal oscillations of Ly6Chi inflammatory monocytes. Science.

[66] Gibbs J.E., Blaikley J., Beesley S., Matthews L., Simpson K.D., Boyce S.H., Farrow S.N., Else K.J., Singh D., Ray D.W. (2012). The nuclear receptor REV-ERBα mediates circadian regulation of innate immunity through selective regulation of inflammatory cytokines. Proc. Natl. Acad. Sci. USA.

[67] Yang G., Wright C.J., Hinson M.D., Fernando A.P., Sengupta S., Biswas C., La P., Dennery P.A. (2014). Oxidative stress and inflammation modulate Rev-erbα signaling in the neonatal lung and affect circadian rhythmicity. Antioxid. Redox Signal..

[68] Griffin P., Dimitry J.M., Sheehan P.W., Lananna B.V., Guo C., Robinette M.L., Hayes M.E., Cedeño M.R., Nadarajah C.J., Ezerskiy L.A. (2019). Circadian clock protein Rev-erbα regulates neuroinflammation. Proc. Natl. Acad. Sci. USA.

[69] Chen H.-C., Chen Y.-C., Wang T.-N., Fang W.-F., Chang Y.-C., Chen Y.-M., Chen I.-Y., Lin M.-C., Yang M.-Y. (2021). Disrupted expression of circadian clock genes in patients with bronchial asthma. J. Asthma Allergy.

[70] Cory T.J., Schacker T.W., Stevenson M., Fletcher C.V. (2013). Overcoming pharmacologic sanctuaries. Curr. Opin. HIV AIDS.

[71] Palmer S., Josefsson L., Coffin J.M. (2011). HIV reservoirs and the possibility of a cure for HIV infection. J. Intern. Med..

[72] Buzon M.J., Massanella M., Llibre J.M., Esteve A., Dahl V., Puertas M.C., Gatell J.M., Domingo P., Paredes R., Sharkey M. (2010). HIV-1 replication and immune dynamics are affected by raltegravir intensification of HAART-suppressed subjects. Nat. Med..

[73] Hatano H., Strain M.C., Scherzer R., Bacchetti P., Wentworth D., Hoh R., Martin J.N., McCune J.M., Neaton J.D., Tracy R.P. (2013). Increase in 2-long terminal repeat circles and decrease in D-dimer after raltegravir intensification in patients with treated HIV infection: A randomized, placebo-controlled trial. J. Infect. Dis..

[74] Twigg H.L., Soliman D.M., Day R.B., Knox K.S., Anderson R.J., Wilkes D.S., Schnizlein-Bick C.T. (1999). Lymphocytic alveolitis, bronchoalveolar lavage viral load, and outcome in human immunodeficiency virus infection. Am. J. Respir. Crit. Care Med..

[75] Nakata K., Weiden M., Harkin T., Ho D., Rom W.N. (1995). Low copy number and limited variability of proviral DNA in alveolar macrophages from HIV-1-infected patients: Evidence for genetic differences in HIV-1 between lung and blood macrophage populations. Mol. Med..

[76] Rose R.M., Krivine A., Pinkston P., Gillis J.M., Huang A., Hammer S.M. (1991). Frequent identification of HIV-1 DNA in bronchoalveolar lavage cells obtained from individuals with the acquired immunodeficiency syndrome. Am. Rev. Respir. Dis..

[77] Devadoss D., Singh S.P., Acharya A., Do K.C., Periyasamy P., Manevski M., Mishra N., Tellez C., Ramakrishnan S., Belinsky S. (2020). Lung Bronchial Epithelial Cells are HIV Targets for Proviral Genomic Integration. bioRxiv.

[78] Chand H.S., Vazquez-Guillamet R., Royer C., Rudolph K., Mishra N., Singh S.P., Hussain S.S., Barrett E., Callen S., Byrareddy S.N. (2018). Cigarette smoke and HIV synergistically affect lung pathology in cynomolgus macaques. J. Clin. Investig..

[79] Chinnapaiyan S., Dutta R.K., Nair M., Chand H.S., Rahman I., Unwalla H.J. (2019). TGF-beta1 increases viral burden and promotes HIV-1 latency in primary differentiated human bronchial epithelial cells. Sci. Rep..

[80] Chinnapaiyan S., Dutta R., Bala J., Parira T., Agudelo M., Nair M., Unwalla H.J. (2018). Cigarette smoke promotes HIV infection of primary bronchial epithelium and additively suppresses CFTR function. Sci. Rep..

[81] Collora J.A., Ho Y.C. (2022). The loud minority: Transcriptionally active HIV-1-infected cells survive, proliferate, and persist. Cell.

[82] Einkauf K.B., Osborn M.R., Gao C., Sun W., Sun X., Lian X., Parsons E.M., Gladkov G.T., Seiger K.W., Blackmer J.E. (2022). Parallel analysis of transcription, integration, and sequence of single HIV-1 proviruses. Cell.

[83] Malone J.L., Oldfield III E.C., Wagner K.F., Simms T.E., Daly R., O’Brian J., Burke D.S. (1992). Abnormalities of morning serum cortisol levels and circadian rhythms of CD4+ lymphocyte counts in human immunodeficiency virus type 1-infected adult patients. J. Infect. Dis..

[84] Lee K.A., Gay C., Byun E., Lerdal A., Pullinger C.R., Aouizerat B.E. (2015). Circadian regulation gene polymorphisms are associated with sleep disruption and duration, and circadian phase and rhythm in adults with HIV. Chronobiol. Int..

[85] Kwon H.-S., Brent M.M., Getachew R., Jayakumar P., Chen L.-F., Schnolzer M., McBurney M.W., Marmorstein R., Greene W.C., Ott M. (2008). Human immunodeficiency virus type 1 Tat protein inhibits the SIRT1 deacetylase and induces T cell hyperactivation. Cell Host Microbe.

[86] Bordoni V., Tartaglia E., Refolo G., Sacchi A., Grassi G., Antinori A., Fimia G.M., Agrati C. (2020). Per2 upregulation in circulating hematopoietic progenitor cells during chronic hiv infection. Front. Cell. Infect. Microbiol..

[87] O’Brien J., Hayder H., Zayed Y., Peng C. (2018). Overview of microRNA biogenesis, mechanisms of actions, and circulation. Front. Endocrinol..

[88] Dutta R.K., Chinnapaiyan S., Unwalla H. (2019). Aberrant microRNAomics in pulmonary complications: Implications in lung health and diseases. Mol. Ther.-Nucleic Acids.

[89] Kozomara A., Birgaoanu M., Griffiths-Jones S. (2019). miRBase: From microRNA sequences to function. Nucleic Acids Res..

[90] Lewis B.P., Burge C.B., Bartel D.P. (2005). Conserved seed pairing, often flanked by adenosines, indicates that thousands of human genes are microRNA targets. Cell.

[91] Kim V.N., Han J., Siomi M.C. (2009). Biogenesis of small RNAs in animals. Nat. Rev. Mol. Cell Biol..

[92] Griffiths-Jones S., Grocock R.J., Van Dongen S., Bateman A., Enright A.J. (2006). miRBase: microRNA sequences, targets and gene nomenclature. Nucleic Acids Res..

[93] Lim L.P., Lau N.C., Garrett-Engele P., Grimson A., Schelter J.M., Castle J., Bartel D.P., Linsley P.S., Johnson J.M. (2005). Microarray analysis shows that some microRNAs downregulate large numbers of target mRNAs. Nature.

[94] Alipoor S.D., Adcock I.M., Garssen J., Mortaz E., Varahram M., Mirsaeidi M., Velayati A. (2016). The roles of miRNAs as potential biomarkers in lung diseases. Eur. J. Pharmacol..

[95] Su B., Fu Y., Liu Y., Wu H., Ma P., Zeng W., Zhang T., Lian S., Wu H. (2018). Potential application of microRNA profiling to the diagnosis and prognosis of HIV-1 infection. Front. Microbiol..

[96] Cox K.H., Takahashi J.S. (2019). Circadian clock genes and the transcriptional architecture of the clock mechanism. J. Mol. Endocrinol..

[97] Hu Y., He T., Zhu J., Wang X., Tong J., Li Z., Dong J. (2021). The link between circadian clock genes and autophagy in chronic obstructive pulmonary disease. Mediat. Inflamm..

[98] Suárez Y., Sessa W.C. (2009). MicroRNAs as novel regulators of angiogenesis. Circ. Res..

[99] Cheng H.-Y.M., Papp J.W., Varlamova O., Dziema H., Russell B., Curfman J.P., Nakazawa T., Shimizu K., Okamura H., Impey S. (2007). microRNA modulation of circadian-clock period and entrainment. Neuron.

[100] Shende V.R., Neuendorff N., Earnest D.J. (2013). Role of miR-142-3p in the post-transcriptional regulation of the clock gene Bmal1 in the mouse SCN. PLoS ONE.

[101] Shende V.R., Goldrick M.M., Ramani S., Earnest D.J. (2011). Expression and rhythmic modulation of circulating microRNAs targeting the clock gene Bmal1 in mice. PLoS ONE.

[102] Tan X., Zhang P., Zhou L., Yin B., Pan H., Peng X. (2012). Clock-controlled mir-142-3p can target its activator, Bmal1. BMC Mol. Biol..

[103] Chaudhuri A.D., Yelamanchili S.V., Marcondes M.C.G., Fox H.S. (2013). Up-regulation of microRNA-142 in simian immunodeficiency virus encephalitis leads to repression of sirtuin1. FASEB J..

[104] Zhang H.-S., Chen X.-Y., Wu T.-C., Sang W.-W., Ruan Z. (2012). MiR-34a is involved in Tat-induced HIV-1 long terminal repeat (LTR) transactivation through the SIRT1/NFκB pathway. FEBS Lett..

[105] Cai X., Hagedorn C.H., Cullen B.R. (2004). Human microRNAs are processed from capped, polyadenylated transcripts that can also function as mRNAs. Rna.

[106] Mazzoccoli G., Colangelo T., Panza A., Rubino R., Tiberio C., Palumbo O., Carella M., Trombetta D., Gentile A., Tavano F. (2016). Analysis of clock gene-miRNA correlation networks reveals candidate drivers in colorectal cancer. Oncotarget.

[107] Hasakova K., Reis R., Vician M., Zeman M., Herichova I. (2019). Expression of miR-34a-5p is up-regulated in human colorectal cancer and correlates with survival and clock gene PER2 expression. PLoS ONE.

[108] Hicks S.D., Khurana N., Williams J., Dowd Greene C., Uhlig R., Middleton F.A. (2018). Diurnal oscillations in human salivary microRNA and microbial transcription: Implications for human health and disease. PLoS ONE.

[109] Zhao X., Zhu X., Cheng S., Xie Y., Wang Z., Liu Y., Jiang Z., Xiao J., Guo H., Wang Y. (2014). MiR-29a/b/c regulate human circadian gene hPER1 expression by targeting its 3′UTR. Acta Biochim. Biophys. Sin..

[110] Landskroner-Eiger S., Qiu C., Perrotta P., Siragusa M., Lee M.Y., Ulrich V., Luciano A.K., Zhuang Z.W., Corti F., Simons M. (2015). Endothelial miR-17∼92 cluster negatively regulates arteriogenesis via miRNA-19 repression of WNT signaling. Proc. Natl. Acad. Sci. USA.

[111] Betel D., Wilson M., Gabow A., Marks D.S., Sander C. (2008). The microRNA. org resource: Targets and expression. Nucleic Acids Res..

[112] Gao Q., Zhou L., Yang S.-Y., Cao J.-M. (2016). A novel role of microRNA 17-5p in the modulation of circadian rhythm. Sci. Rep..

[113] Triboulet R., Mari B., Lin Y.-L., Chable-Bessia C., Bennasser Y., Lebrigand K., Cardinaud B., Maurin T., Barbry P., Baillat V. (2007). Suppression of microRNA-silencing pathway by HIV-1 during virus replication. Science.

[114] Nagel R., Clijsters L., Agami R. (2009). The miRNA-192/194 cluster regulates the Period gene family and the circadian clock. FEBS J..

[115] Na Y.-J., Sung J.H., Lee S.C., Lee Y.-J., Choi Y.J., Park W.-Y., Shin H.S., Kim J.H. (2009). Comprehensive analysis of microRNA-mRNA co-expression in circadian rhythm. Exp. Mol. Med..

[116] Witwer K.W., Watson A.K., Blankson J.N., Clements J.E. (2012). Relationships of PBMC microRNA expression, plasma viral load, and CD4+ T-cell count in HIV-1-infected elite suppressors and viremic patients. Retrovirology.

[117] Zhou L., Miller C., Miraglia L.J., Romero A., Mure L.S., Panda S., Kay S.A. (2021). A genome-wide microRNA screen identifies the microRNA-183/96/182 cluster as a modulator of circadian rhythms. Proc. Natl. Acad. Sci. USA.

[118] Feng Y.-Z., Yu Y., Zhou Y.-F., Yang Y.-W., Lei M.-Q., Lian J.-P., He H., Zhang Y.-C., Huang W., Chen Y.-Q. (2020). A natural variant of miR397 mediates a feedback loop in circadian rhythm. Plant Physiol..

[119] Chen R., D’Alessandro M., Lee C. (2013). miRNAs are required for generating a time delay critical for the circadian oscillator. Curr. Biol..

[120] Yoo S.-H., Kojima S., Shimomura K., Koike N., Buhr E.D., Furukawa T., Ko C.H., Gloston G., Ayoub C., Nohara K. (2017). Period2 3′-UTR and microRNA-24 regulate circadian rhythms by repressing PERIOD2 protein accumulation. Proc. Natl. Acad. Sci. USA.

[121] Chinnapaiyan S., Dutta R.K., Devadoss D., Chand H.S., Rahman I., Unwalla H.J. (2020). Role of non-coding RNAs in lung circadian clock related diseases. Int. J. Mol. Sci..

[122] Datta Chaudhuri A., Yelamanchili S.V., Fox H.S. (2013). MicroRNA-142 reduces monoamine oxidase A expression and activity in neuronal cells by downregulating SIRT1. PLoS ONE.

[123] Dutta R.K., Chinnapaiyan S., Santiago M.J., Rahman I., Unwalla H.J. (2021). Gene-specific MicroRNA antagonism protects against HIV Tat and TGF-beta-mediated suppression of CFTR mRNA and function. Biomed Pharm..

